# Atypical manifestations of Dengue fever: case series in tertiary care hospital in Nepal

**DOI:** 10.1093/bjrcr/uaaf038

**Published:** 2025-07-18

**Authors:** Sudeep KC, Himani Poudyal

**Affiliations:** Department of Radiology, Patan Academy of Health Sciences, Lalitpur, Kathmandu, 26500, Nepal; Department of Internal Medicine, Dhulikel Hospital, Kavre, 11008, Nepal

**Keywords:** cerebral edema, dengue fever, hemorrhagic infarct, meningoencephalitis

## Abstract

Dengue fever is common in Southeast Asia, including Nepal, caused by the Flavi virus transmitted through mosquito bites of *Aedes aegypti* species. Symptoms include high-grade fever, skin rash, headache and arthralgia, with a low case fatality rate of less than 1%. Severe forms are characterized by low platelet count, vascular leakage, and low blood pressure, often leading to life-threatening complications. Common imaging findings include gall bladder wall thickening, hepatosplenomegaly, ascites, pericardial effusion, and pleural effusion. Dengue was initially considered non-neurotropic, but recent studies suggest that the virus can invade the central nervous system, indicating its neurotropic potential presenting with encephalitis and meningitis. In this case series, we have described atypical imaging findings of 7 patients in patients with laboratory confirmed dengue fever, which revealed imaging features of psoas hematoma in 1 case, diffuse pulmonary haemorrhage in 1 case, multifocal pneumonia in 1 case, hemorrhagic stroke with venous thrombosis in 1 case, dengue meningoencephalitis in 2 cases and dengue encephalitis with Cytotoxic lesion of the corpus callosum in 1 case. This case series emphasizes the important role of imaging findings in severe dengue patients with suspicion of unusual complications as early detection and prompt treatment are crucial for recovery and to prevent fatal complications.

## Introduction

In Nepal, dengue is considered endemic and falls under the endemicity category A as classified by World Health Organization (WHO), indicating a significant public health concern.^1^ Symptomatic patients may present with dengue fever, undifferentiated viral fever, or dengue hemorrhagic fever (DHF). The year 2022 witnessed Nepal’s largest dengue outbreak, with a total of 54,784 reported cases and 88 fatalities.^2^ To address this escalating trend, it is crucial to implement effective prevention strategies, ensure prompt diagnosis, provide appropriate treatment, and manage complications. While radiology does not play a direct role in confirming the diagnosis of dengue, it does play a vital role in identifying atypical manifestations, predicting severity, and assessing unexpected manifestations of severe dengue.^3^ Most published literature on Dengue focuses mainly on neurological manifestation of dengue, and only few articles focus on multi system imaging findings.^4^

## Case presentation

### Case 1

A 73-year-old male arrived at the Emergency Department complaining of fever, muscle pain, and multiple episodes of loose stool with melena over the past 2 days. Upon admission, the patient had a high-grade fever accompanied by tachycardia. Systemic examination was normal. However, laboratory results indicated a significantly low platelet count of 13,000/mm^3^, PT/INR of 12.5/1.0, and Dengue NS1 was positive. Ultrasonography (USG) of abdomen revealed mild hepatomegaly with minimal ascites and mild thickening of the gall bladder wall. Patient was then admitted in the intensive care unit (ICU) and was started on conservative treatment with antipyretics, intravenous fluids and platelet-rich plasma.

**Figure 1.**
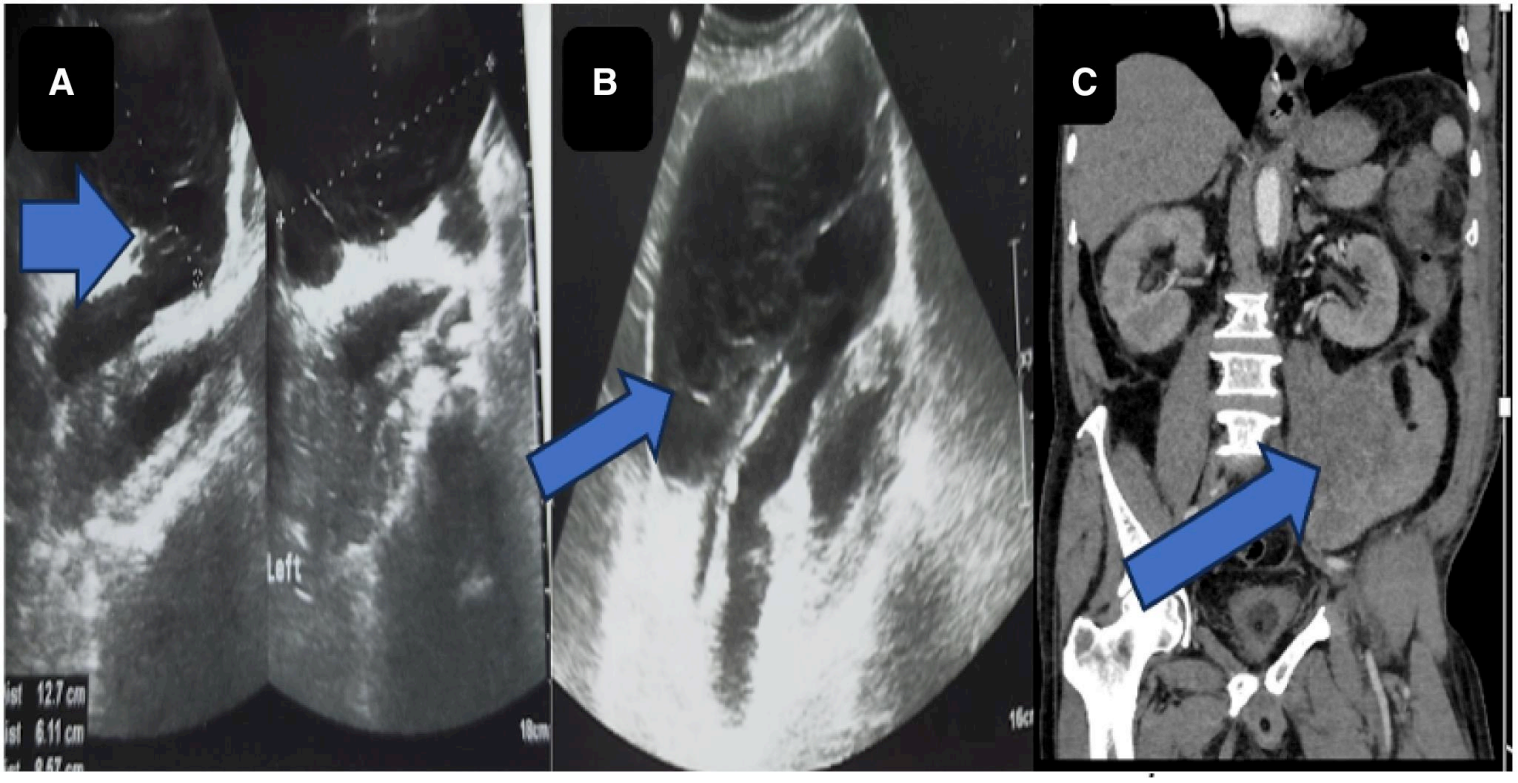
Ultrasound images (A, B) showing well-defined hypodense collection measuring 12.7 × 6.1 × 8.5 cm with echogenic contents and multiple strands in the left psoas muscle suggestive of intra muscular hematoma. Coronal sections of contrast-enhanced CT abdomen (C) showing large hematoma involving the intramuscular plane of posterolateral aspect of psoas muscle.

On the fifth day of admission, the patient complained of lower abdominal pain and abdominal distension and lower abdominal pain radiating to the back. During physical examination, the patient had pallor, distended abdomen with significant tenderness, and sluggish bowel sounds. Blood investigations revealed a total count of 5500/mm^3^, Hb of 7.2%, and falling platelet count of 10 000/mm^3^. Repeat USG of abdomen showed a heteroechoic lesion along the left psoas muscle (Figure 1A and B), while a contrast-enhanced CT (CECT) scan confirmed the presence of a large left psoas hematoma (Figure 1C).

No active bleeding was seen within the hematoma. Hence, conservative management was continued with close monitoring. The psoas muscle hematoma gradually resolved in later imaging studies. After a smooth recovery, the patient was discharged from the hospital after 2 weeks. Follow-up imaging after 6 months showed complete resolution of the collection.

### Case 2

**Figure 2.**
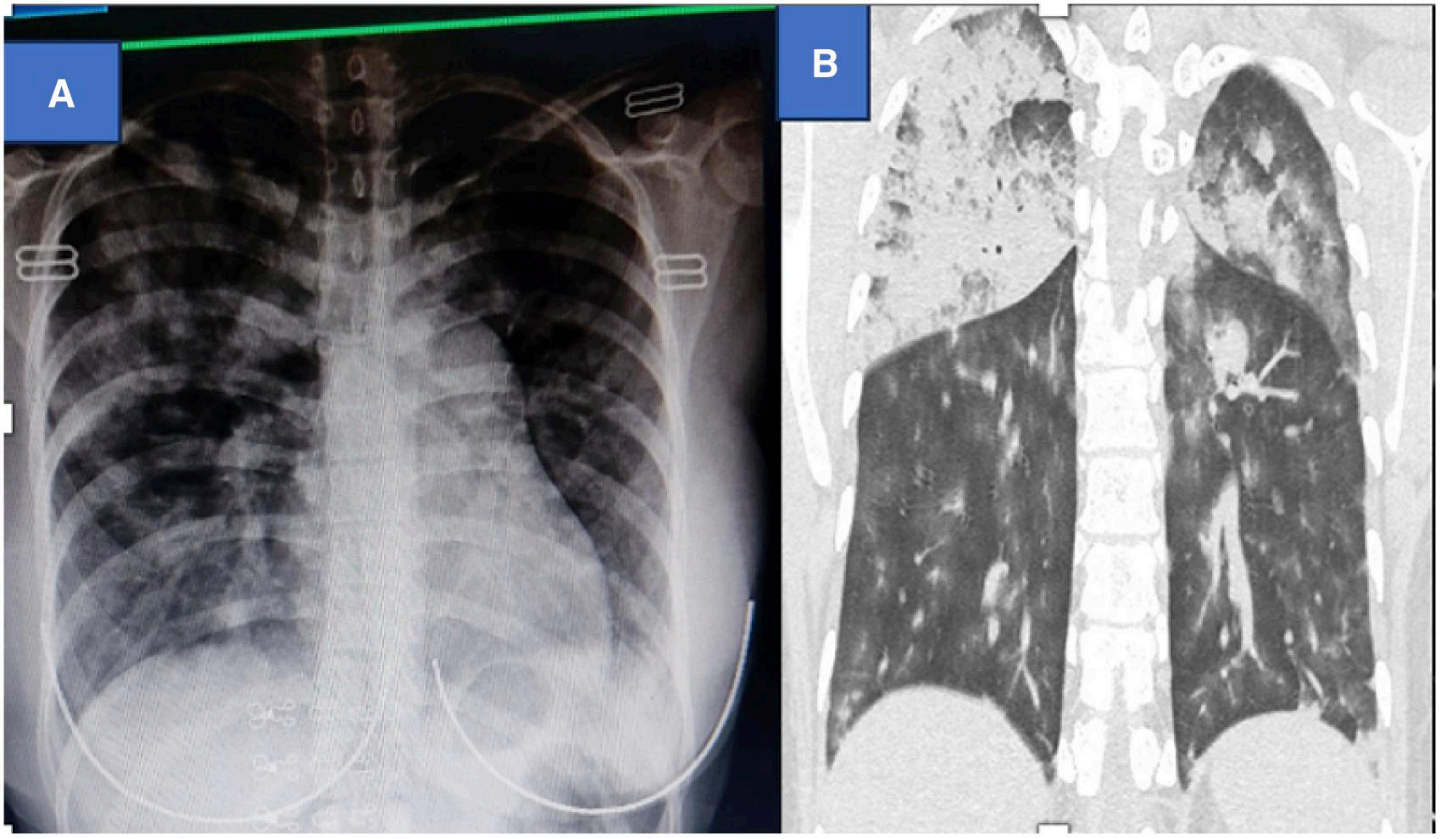
Chest X-ray showed diffuse patchy airspace opacities in the bilateral lung field (Figure A). Figure (B) coronal HRCT showed confluent ground glass opacities with air bronchogram in bilateral upper lobe (right > left); predominant air space opacities in bilateral upper lobe of lung.

A 21-year-old female patient presented to the Emergency Department with hemoptysis for 1 day, fever and shortness of breath for 1 week. She had low blood pressure, high respiratory rate, and low oxygen saturation (BP: 90/60 mm hg, RR: 28, SpO2: 50% in RA, 86% in reservoir). Examination revealed crackles in both lung fields. Blood investigation showed a reduced platelet count of 100 000/mm^3^. Covid antigen was negative, but a positive dengue NS1 test was positive. Chest X-ray showed bilateral pulmonary infiltrates (Figure 2A). HRCT chest showed collapse/consolidation in bilateral lungs predominantly in the upper lobe; high-density area in the right lung, which was relatively hyperdense than that of the aorta in the narrow window setting (Figure 2B). The case was provisionally diagnosed as Diffuse pulmonary alveolar haemorrhage. The patient was treated with antibiotics, antipyretics, oxygen, and nebulization. Clinically and haemodynamically patient showed improvement and was discharged after 2 weeks.

**Figure 3.**
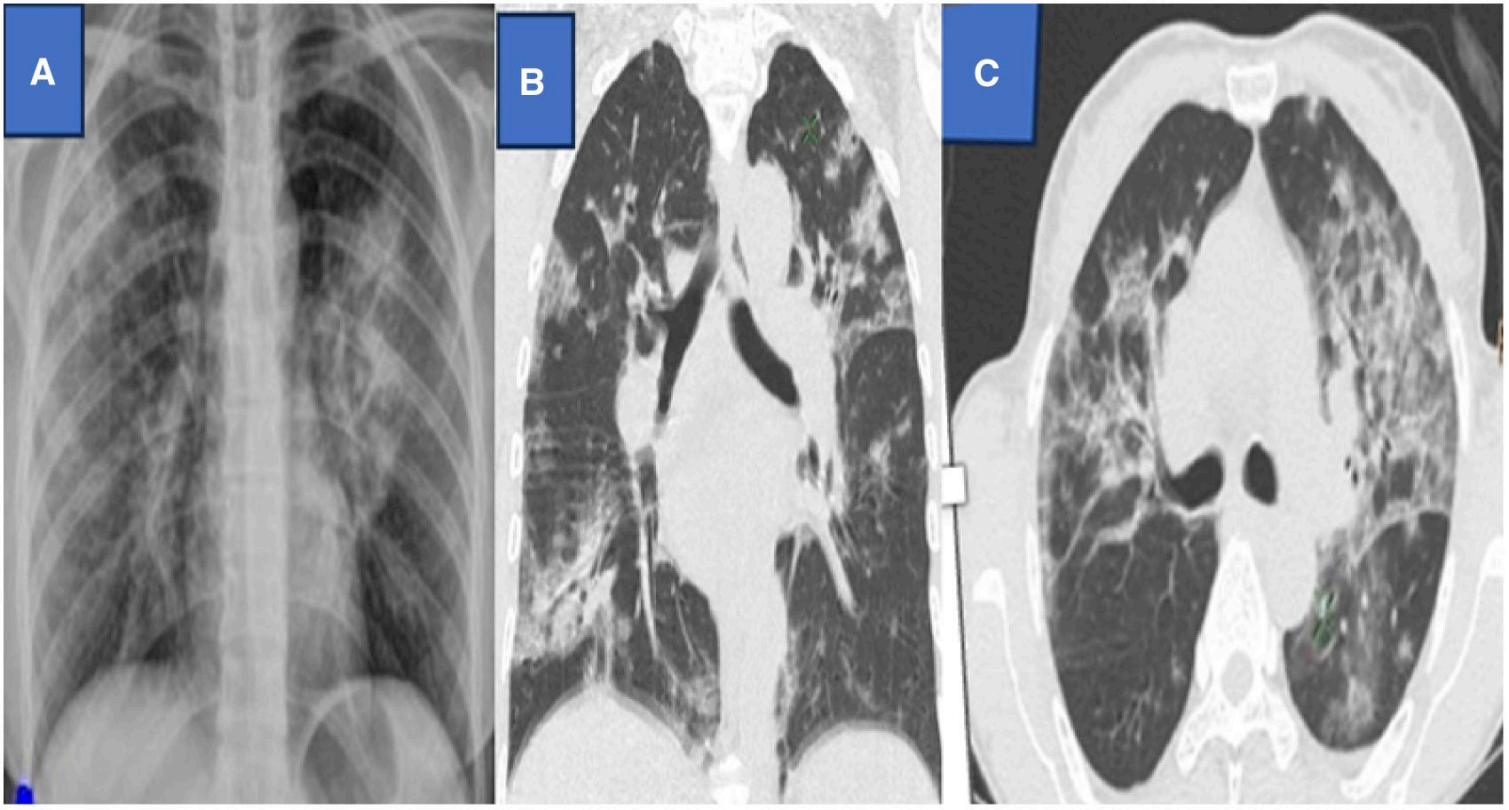
Chest X-ray (A) showed bilateral multifocal areas of airspace opacities. HRCT images (B and C) showing areas of bilateral multifocal ground-glass opacities and consolidation.

**Figure 4.**
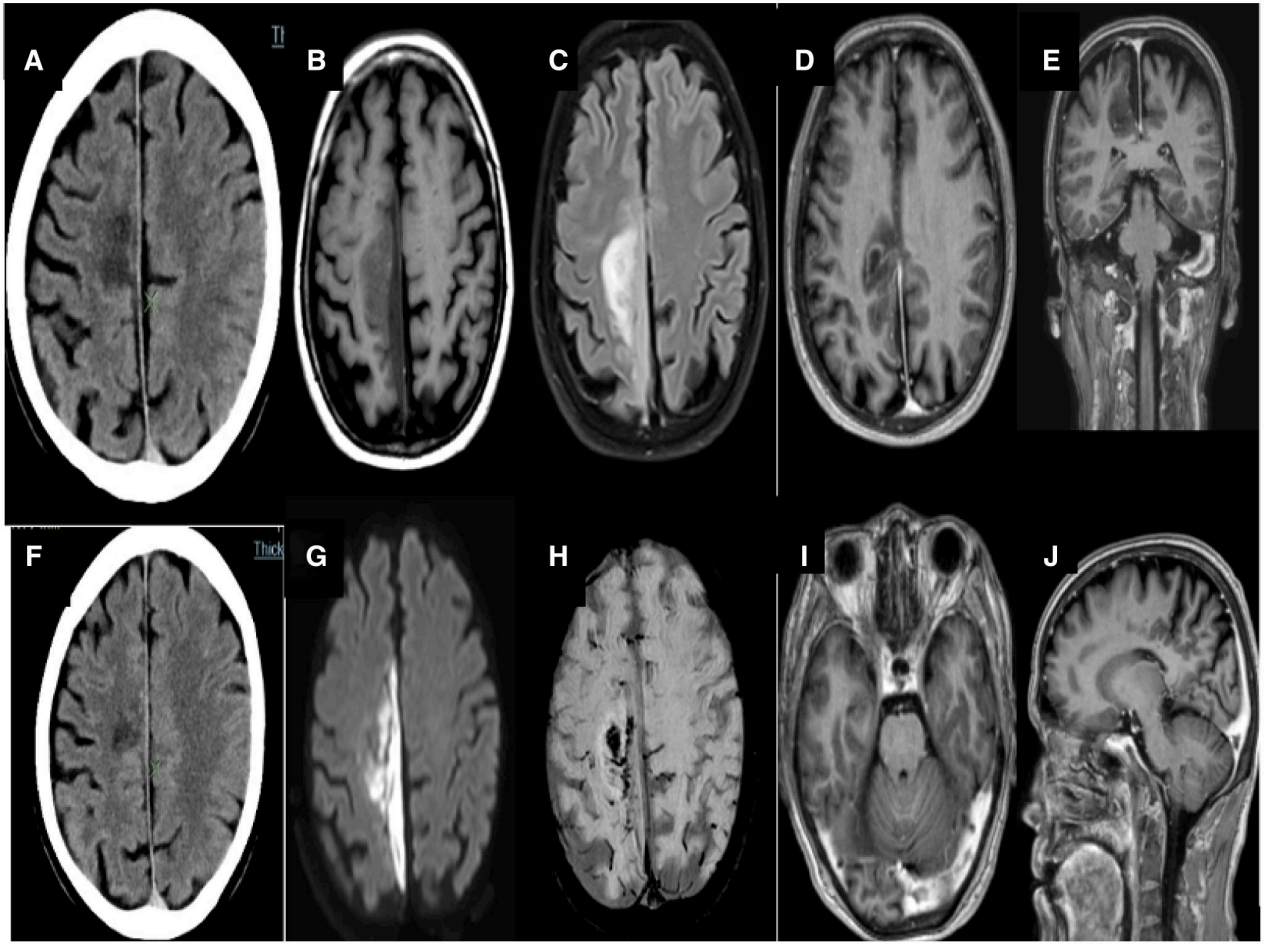
Axial CT showed ill-defined hypodense areas in the right para-falcine region without haemorrhagic component (A, F). Magnetic resonance images of the brain (B). T1-weighted axial image shows low signal intensity involving the right para-falcine region with corresponding high signal in T2-weighted axial image (C). Post contrast study shows minimal peripheral enhancement (D). Diffusion-weighted image (G) show high signal intensity involving the right parasagittal region with gyral haemorrhage (H). Post-gadolinium T1- weighted image displays a filling defect in the left sigmoid sinus (E, I, J).

**Figure 5.**
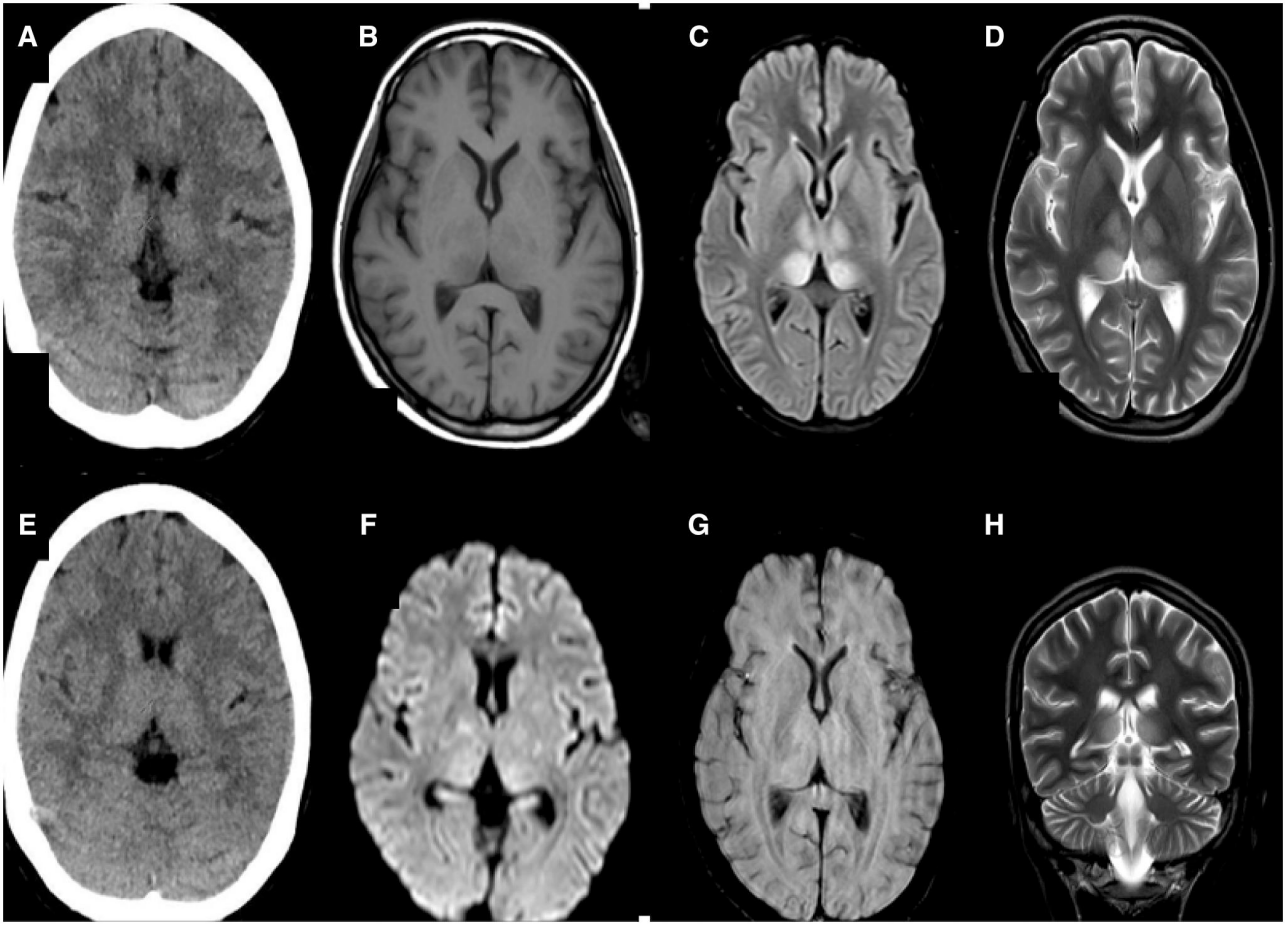
Axial CT showed subtle symmetrical hyperdensity in bilateral thalami. No hemorrhagic component seen (A and E). Subtle T1 hypointensity (B) in bilateral thalami. The corresponding lesion appears hyperintense T2/FLAIR hyperintense (C, D). Subtle diffusion restriction seen with high signal on DWI (F). No hemorrhagic component seen in SWI images (G).

### Case 3

A 54-year female presented to the Emergency Department with a history of fever and cough for 5 days. Fever was associated with headache, retroorbital pain, and shortness of breath. On systemic chest examination, bilateral diffuse crepitations were heard. Th rest of the systemic examinations were normal. Blood investigation showed reduced platelet count of 87 000/mm^3^. Dengue NS1 was positive. Chest X-ray showed ill-defined areas of air-space opacities in bilateral lungs, and HRCT chest showed multifocal ill-defined areas of air-space opacities in bilateral sides suggestive of multifocal pneumonia as shown in Figure 3. Patient was managed with antibiotics, nebulization, and oxygen. The patient gradually recovered with maintained oxygen saturation in room air and was discharged after 2 weeks.

### Case 4

A 65-year male was presented to the Emergency Department with complaints of weakness of the left side of the body for 1 day associated with a high-grade fever, headache, and myalgia. Neurological examination showed reduced power of the left lower limb. The rest of the systemic examinations were within the normal limit. Lab investigations were within the normal limit except for Dengue-IgM positive.

A CT scan of the head showed a hypodense area in the right parafalcine region and right parietal lobe as shown in Figure 4 (A and F). No hemorrhagic component was seen. Also, elliptical hypodense extra axial collection was seen along the adjacent right falcine region with slight displacement of the cortex (Figure 4B). Urgent CE MRI of the brain was done, which revealed T2/FLAIR hyperintensities in the right parafalcine region with diffusion restriction and blooming on gradient sequence (GRE) (Figure 4D, E, I, J). Postcontrast images show peripheral enhancement around a hypointense area in the right parafalcine region and filling defect in left sigmoid sinus as shown in Figure 4 (I and J). Considering his clinical, laboratory, and imaging findings, the diagnosis of right fronto-parietal haemorrhagic stroke with acute right parafalcine subdural hematoma and left sigmoid sinus thrombosis was made. The patient was then conservatively managed and was discharged after 3 weeks. Physiotherapy was continued where gradual recovery of his neurologic functions was seen after a 1-year follow-up.

### Case 5

A 15-year-old female patient presented with altered sensorium for a day accompanied by fever and chills/rigor for a week. Clinical examination revealed neck rigidity and a Glasgow Coma Scale (GCS) of E4V1M6 (11/15) with positive Nystagmus, Hypertonia, Bilateral hyperreflexia, and positive Babinski sign. Kernig’s and Brudzinski sign were also positive. Initially, tests for Dengue, Brucella, Leptospira, and scrub typhus were negative. Her routine hemogram revealed lymphocytosis. A non-contrast computed tomography scan revealed subtle hyperdensity in the bilateral thalami as shown in Figure 5 (A and E) Non-contrast MRI of the brain showed T2/FLAIR high signal intensity in the bilateral thalami without haemorrhagic component, suggesting meningoencephalitis (Figure 5B–D, F, G). Blood and cerebrospinal fluid (CSF) cultures showed no growth. Subsequently, a CSF study indicated the possibility of viral meningitis. She tested positive for NS1 Antigen for Dengue on the third day. The final diagnosis was viral meningoencephalitis. The patient was managed conservatively and made a complete recovery. She was discharged after 1 week and is under regular follow-up in an outpatient clinic.

### Case 6

**Figure 6.**
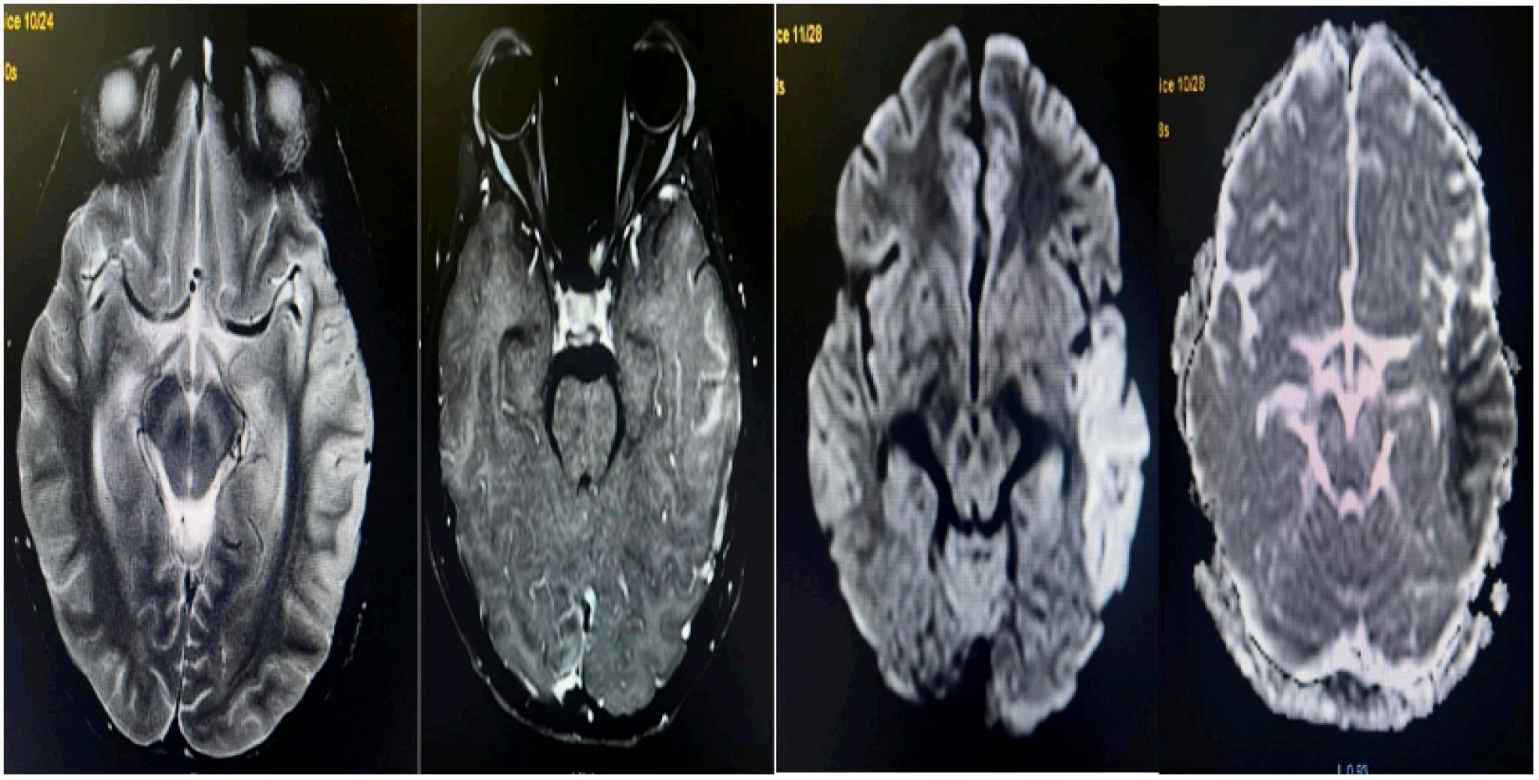
Axial T2 cortical hyperintensity in the left temporal region with gyriform enhancement in post-contrast T1 fat sat image. Corresponding areas show diffusion restriction with high signal on DWI with a low signal on ADC map.

A 36-year-old female was presented to the Emergency Department with a 5-day history of fever and headache. She tested positive for Dengue NS1, and the platelet count was 80 000/mm^3^. On the second day of admission, she developed weakness on the right side of her lower limb and slurring of speech. CE MRI brain revealed bilateral symmetrical areas of altered signal intensity involving the left parieto-temporal lobe, which were hyperintense on FLAIR and T2 and hypointense on T1‐weighted sequence with diffusion restriction. Post-contrast study showed meningeal enhancement. No blooming on GRE was seen (Figure 6). The patient was then managed conservatively with a diagnosis of dengue meningoencephalitis. The patient was discharged after 1 week and was kept in regular follow-up. Physiotherapy was continued; the patient made slow but steady neurological recovery.

**Figure 7.**
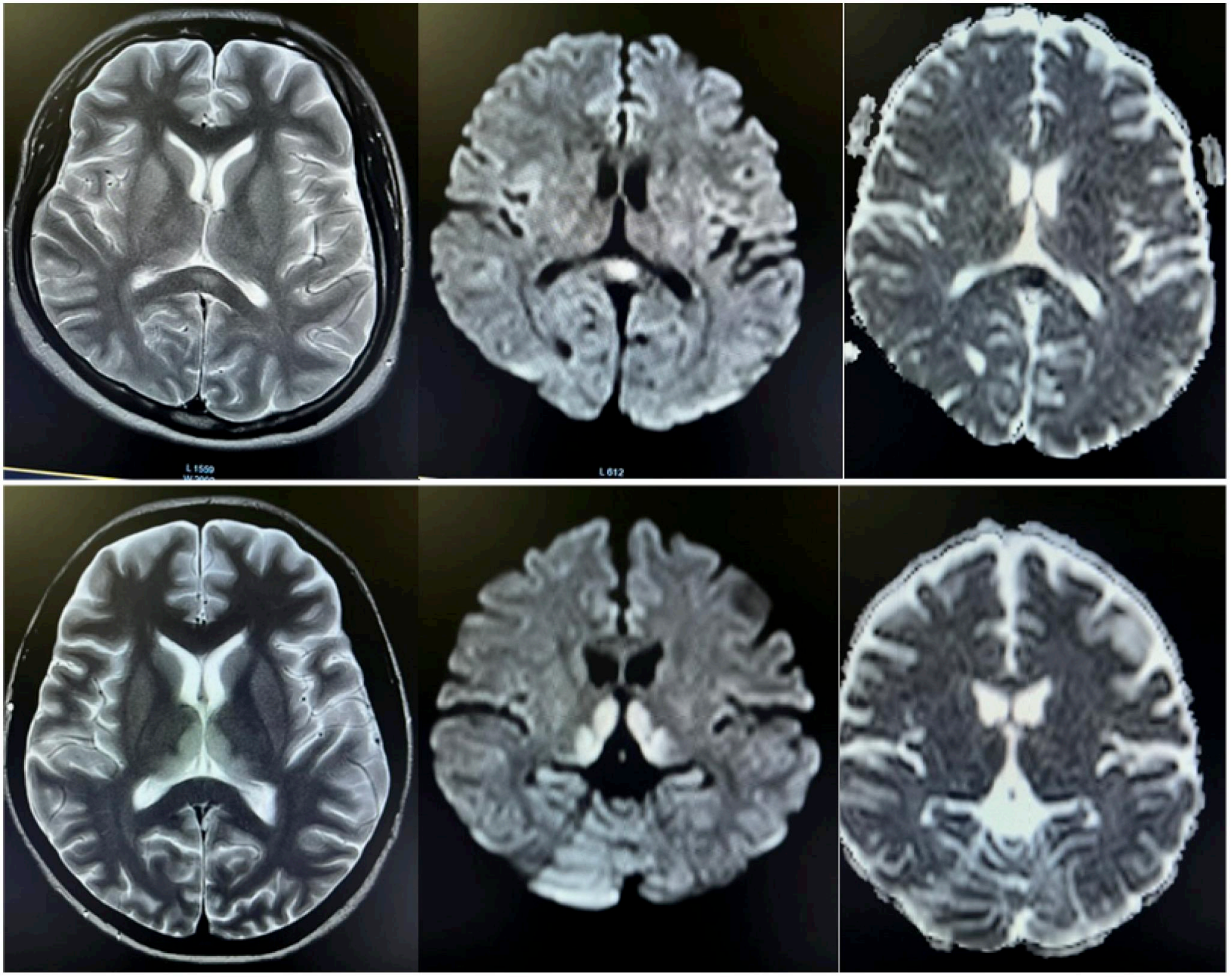
Small well-defined ovoid lesion in the midline within the splenium of the corpus callosum and bilateral thalami and high signal on T2 and FLAIR with no enhancement on postcontrast sequence. The DWI/ADC sequence shows mild restricted diffusion.

### Case 7

A 23-year-old female presented to the Emergency Department with 1 episode of generalized tonic-clonic seizure and 1-week history of fever, headache, and dizziness. Systemic examination was unremarkable. Laboratory investigations showed a positive for Dengue NS1, while the rest of the lab results were normal. To rule out dengue meningoencephalitis, a contrast-enhanced MRI of the brain was performed on the fifth day of the fever. The MRI revealed symmetrical T2/FLAIR hyperintensities and corresponding T1 hypointensity in the thalami and splenium of the corpus callosum, and showed minimal enhancement with contrast administration as shown in Figure 7. These areas also showed diffusion restriction (DWI), but no blooming was observed on the GRE (Figure 7). The patient was diagnosed with dengue encephalitis and Cytotoxic lesion of the corpus callosum. She received conservative treatment for one week and was discharged on day 7 after complete neurological recovery.

## Discussion

WHO categorizes dengue into 3 groups: without warning signs, with warning signs, and severe dengue. This updated classification includes severe complications affecting organs like the liver, heart, and central nervous system.^5^ There are 4 serotypes of the dengue virus: DENV-1, DENV-2, DENV-3, and DENV-4. Immunity to one serotype does not protect against the others. When infection is caused by multiple serotypes or is associated with secondary infections, the severity is significantly increased.^2^ As the burden of the disease continues to rise, there has been an increase in atypical manifestations, including neurological, hepatic, renal, and other organ dysfunction. The initial phase includes fever, headache, joint pain, and rash for 3-7 days. The critical phase lasts 24-48 hours with plasma leaks, polyserositis, hypovolemia, bleeding, and multi-organ involvement. The final phase, convalescent or resolution phase, lasts 2-5 days with fluid reabsorption.^1^

### Bleeding disorder

The hemorrhagic symptoms in dengue fever are due to several factors, such as low platelet count, increased fragility of blood vessels, impaired platelet function, and accelerated breakdown of blood clots. Even in milder cases of dengue, minor haemorrhagic symptoms like petechiae, purpura, nosebleeds, and bleeding gums are common and can be seen in around one-third of cases.^6^

In our case, psoas hematoma may be due to low platelet count. Identifying muscle hematomas and monitoring vital signs is crucial to prevent shock. A sudden decline in haemoglobin and hematocrit levels without external bleeding or painful swelling in an unusual location should prompt investigation for occult haemorrhage using imaging studies. Daily monitoring of haemoglobin, hematocrit, and platelet count is vital as diagnosis solely based on symptoms is challenging.

### Central nervous system involvement

Recent research and case studies have revealed the neurotropic potential of the dengue virus, challenging previous assumptions that it was non-neurotropic.^7^ Our case series highlights various neurological complications, including encephalopathy, encephalitis, meningitis, and stroke. Other complications, including acute disseminated encephalomyelitis (ADEM), Guillain-Barré syndrome, intracranial haemorrhage, subdural hematoma, and optic neuritis are commonly associated with dengue. Encephalitis and meningitis occur when the virus directly invades the CNS, causing brain inflammation. Encephalopathy, on the other hand, is typically a result of systemic disturbances.^8^

Patients with dengue-related encephalitis may show various neurological symptoms, including mental status changes, headaches, seizures, nystagmus, focal deficits, and vertigo. The basal ganglia, thalamus, white matter, gray matter, brainstem, and cerebellum are most affected. Imaging studies often reveal specific abnormalities and can be seen in other types of encephalitis, so serological testing may be necessary for accurate diagnosis.^9^^,^^10^ The dengue virus is associated with micro-haemorrhages in above mentioned regions.

MRI is more sensitive than CT for confirming dengue encephalitis and pinpointing the affected areas. Lesions show restricted diffusion on DWI, hemorrhagic foci on SWI, and minimal enhancement on contrast-enhanced images. The “dengue double doughnut sign” on DWI and the “inverted dengue double doughnut sign” on postcontrast T1 fat saturation sequences are unique to dengue encephalitis involving the thalamus.^11^

Differential diagnoses include Japanese encephalitis, herpes simplex encephalitis, and ADEM. Japanese encephalitis affects the basal ganglia-thalamus complex bilaterally, while herpes simplex encephalitis impacts the temporal/basifrontal lobes. Hemorrhagic regions are common in herpes encephalitis but rare in Japanese encephalitis and ADEM.^12^ Cerebellar involvement in dengue patients may be linked to an immune-mediated process post-infection. Subarachnoid, extradural, and subdural haemorrhage (SDH) have also been reported with dengue infection.^11^^,^^13^

Multiple studies have examined MRI findings in dengue encephalitis. Weerasinghe and Medagam^14^ reported a case of meningoencephalitis with changes in white and gray matter visible on T2W and FLAIR images. Kumar et al^15^ introduced the “double doughnut sign” in 2017, based on specific thalamic MRI patterns. Garg et al.^16^ presented a case of cortical laminar necrosis in a 12-year-old male with fever and impaired consciousness.

Shah et al^17^ reported a dengue double doughnut sign in their DWI study as well. Our findings were consistent with those of Bhoi et al^18^ who identified T2 and FLAIR hyperintensity along with diffusion restriction, while Borawake et al^19^ mentioned petechial haemorrhages in their cases, similar to our case.

### Gastro-intestinal involvement

Common signs include ascites and gastrointestinal bleeding, as well as unexpected surgical emergencies such as acute acalculous cholecystitis, appendicitis, and pancreatitis have been observed in dengue fever.^20^ Recognizing these varied presentations is crucial to prevent unnecessary surgeries and reduce the risks of complications and death. Hepatitis in dengue fever is caused by the virus affecting liver cells, while fulminant hepatic failure is associated with shock and disseminated intravascular coagulation in severe cases. Acalculous cholecystitis is characterized by gallbladder mucosal swelling and pericholecystic fluid without gallstones as seen in our case 1. In rare instances, acute pancreatitis may result from the virus’s impact or an autoimmune response triggered by molecular mimicry.^21^

Shamim et al^22^ study on 357 dengue patients revealed that most had nonspecific abdominal pain, while a small percentage (12.4%) had acute abdominal pain. A similar finding was also seen by Khor et al^23^ among patients with DHF or dengue shock syndrome. Study conducted by Ooi et al^24^ concluded that certain symptoms (abdominal pain and tenderness, gastrointestinal bleed, jaundice, hepatomegaly, and ascites) could help identify dengue patients in need of intensive care. Our first case required admission in ICU with constant monitoring for the complications.

### Respiratory system involvement

Severe cases of DHF often lead to pulmonary complications, such as pleural effusions, acute respiratory distress syndrome (ARDS), pneumonitis, and pulmonary haemorrhage.^25^ These complications are typically linked to, which causes increased vascular permeability. The most common chest imaging abnormality in dengue is bilateral pleural effusion, with unilateral effusion more likely to occur on the right side. Other parenchymal abnormalities like ground-glass opacities, consolidations, interlobular septal thickening, and nodules indicating oedema or pulmonary haemorrhage.^25^^,^^26^ Diffuse alveolar haemorrhage is rare but can be associated with severe, often fatal, forms of the disease. Hemoptysis is reported in 1.4% of dengue infections. Severe cases of dengue are more likely to have extensive pulmonary complications compared to milder cases, potentially serving as an indicator of a severe presentation of the disease in patients.^27^

The findings are in line with the study by Lum et al.^28^ which have demonstrated that DHF can lead to the development of ARDS Moreover, Venkata and Krishnan have observed that among the 56 patients at fifth and seventh day of fever, 55 displayed right-sided pleural effusion, while 37 had left-sided pleural effusion.^29^ This similar finding was also observed in systemic review by Kaagaard et al and suggested 1/3 of dengue patients presented with pleural effusion and the frequency increased with severity and younger age.^30^

### Other systemic involvement

Acute renal failure is mainly due to shock-induced acute tubular necrosis. It could also be due to multi organ dysfunction syndrome (MODS) and rhabdomyolysis.^31^ None of our case reported acute renal failure. Cardiac manifestations such as conduction blocks, atrial fibrillation, and ectopic ventricular beats are due to myocarditis.^32^ There were no cardiac manifestations in our case series.

## Conclusion

Dengue fever, traditionally diagnosed based on clinical and laboratory findings, can present with atypical radiological manifestations that indicate severe systemic involvement. This case series highlights rare but significant imaging findings, such as psoas hematoma, pulmonary haemorrhage, multifocal pneumonia, meningoencephalitis, and venous thrombosis. Recognizing these atypical features is crucial for early diagnosis, timely intervention, and appropriate management, especially in endemic country where the case load is very high. Further studies are needed to understand the prognostic implications of these imaging abnormalities and their impact on patient outcomes.

## Learning points

1. Atypical manifestations of dengue are not uncommon highlighting the importance of recognizing severe dengue beyond classical symptoms. These complications require early suspicion, close monitoring, and timely supportive management to reduce morbidity and mortality.
2. Diagnosis can be challenging due to non-specific symptoms, but imaging can help in assessing the extent of organ involvement, guide interventions, and differentiate dengue-related complications from other conditions.

## Data Availability

Any required information is available upon request from the corresponding author.
